# Converging Representations of Attention-Deficit/Hyperactivity Disorder and Autism on Social Media: Linguistic and Topic Analysis of Trends in Reddit Data

**DOI:** 10.2196/70914

**Published:** 2025-05-20

**Authors:** Jemima Kang, Nick Haslam, Mike Conway

**Affiliations:** 1 School of Computing and Information Systems University of Melbourne Melbourne Australia; 2 Melbourne School of Psychological Sciences University of Melbourne Melbourne Australia

**Keywords:** ADHD, autism, Reddit, social media, natural language processing, machine learning

## Abstract

**Background:**

Social media platforms have witnessed a substantial increase in mental health–related discussions, with particular attention focused on attention-deficit/hyperactivity disorder (ADHD) and autism. This heightened interest coincides with growing neurodiversity advocacy. The impact of these changes in the conceptualization of ADHD and autism, and the relationship between the 2 conditions, remains underexplored.

**Objective:**

We aim to characterize and understand how the relationship between ADHD and autism has evolved in public discourse over the past decade and explore reasons for their growing alignment.

**Methods:**

Using Reddit data from 2012 to 2022, we investigated the frequency of ADHD mentions in r/autism and autism mentions in r/ADHD, compared to commonly mentioned conditions. We analyzed user overlap between the 2 subreddits to track cross-subreddit discussions. Following this, we assessed changes in semantic similarity between ADHD and autism using Word2Vec embedding models, alongside commonly mentioned conditions. Finally, thematic changes in subreddit discussions were explored using BERT-based topic modeling across 2 time periods.

**Results:**

Our analysis revealed that ADHD and autism have become progressively more associated across these multiple dimensions. In r/ADHD, there was a steep rise in the proportion of posts mentioning “autism” in 2021, overtaking “bipolar” and “OCD” (obsessive-compulsive disorder) to become the most frequently mentioned condition. Similarly, ADHD mentions increased steadily in r/autism, while the frequency of posts mentioning “OCD,” “PTSD” (posttraumatic stress disorder), and “bipolar” remained stable and low. User overlap between these subreddits grew substantially beginning in 2020. Semantic analysis showed ADHD and autism becoming more closely related from 2019 onward, compared to other conditions. Last, topic modeling indicated growing thematic convergence in ADHD- and autism-related discussions, which reflected an increasing shared emphasis on the experiences of adults with ADHD and autism, challenges in accessing diagnostic assessments, and interpersonal difficulties.

**Conclusions:**

Our study clarifies how discourse around these 2 conditions has converged during a period when they have both attracted rising public attention. These findings contribute to wider discussions about the impacts of rising public interest in mental health concepts. They illustrate that public understandings of relationships between conditions are dynamic and changing in ways that diverge from diagnostic frameworks. Future research should continue investigating changing mental health conceptualizations on social media, as these dynamics are becoming increasingly important for the future of psychiatric practice.

## Introduction

### Background

The rising demand for attention-deficit/hyperactivity disorder (ADHD) and autism spectrum disorder (hereafter autism) assessments has placed clinical services under significant strain in recent years [1]. One driving factor of this trend is the growing recognition of co-occurring ADHD and autism, particularly following the introduction of the *Diagnostic and Statistical Manual of Mental Disorders, Fifth Edition* (*DSM-5*), which for the first time permitted them to be diagnosed together. This change prompted greater awareness of the links between the 2 conditions among clinicians and health care providers as well as the public. However, research investigating shifts in the relationship between ADHD and autism has primarily focused on its clinical and epidemiological aspects [2], overlooking how it is understood in the wider culture.

### Brief Diagnostic History of Autism and ADHD

Conceptualizations of ADHD and autism have evolved as scientific knowledge has advanced and diagnostic systems have been revised. Autism was listed as a symptom of child schizophrenia in the first 2 editions of the *Diagnostic and Statistical Manual of Mental Disorders* (*DSM*), classified under the broader category of “pervasive developmental disorders” in *Diagnostic and Statistical Manual of Mental Disorders, Third Edition* (*DSM-III*; 1980) with subtypes including Asperger syndrome, and redefined as the unified diagnosis of autism spectrum disorder in *DSM-5* [3]. Autism is currently understood to be characterized by social communication challenges, restricted interests, and repetitive behaviors [4].

ADHD has undergone a similar evolution. It was initially termed “hyperkinetic reaction of childhood” in the *Diagnostic and Statistical Manual of Mental Disorders, Second Edition,* (*DSM-II*; 1968), which emphasized symptoms of hyperactivity and distractibility. Subsequent editions shifted focus to an attentional deficit. *DSM-III* (1980) renamed it “attention-deficit disorder,” which *DSM-III-R* (1987) changed to “attention-deficit/hyperactivity disorder” (ADHD). The *DSM-5* (2013) expanded the diagnosis so that it could be applied to adolescents and adults rather than children alone. ADHD is currently understood to be characterized by inattention, hyperactivity, and impulsivity [4].

The relationship between autism and ADHD has also changed in recent decades. Evidence of high comorbidity [5] and symptom overlap [6] led to *DSM-5* allowing ADHD and autism to be diagnosed together [7], whereas ADHD had been an exclusion criterion for autism in *Diagnostic and Statistical Manual of Mental Disorders, Fourth Edition* (*DSM-IV*) [8]. Since this diagnostic change, a growing body of literature has focused on understanding clinically converging presentations of ADHD and autism [9]. A recent systematic review by Zhong and Porter [10] found that the prevalence of autism in individuals with ADHD ranged from 15% to 64.3%. A meta-analysis by Rong et al [11] estimated that the prevalence of ADHD in individuals with autism is 38.5%, with 40.2% of individuals with autism experiencing ADHD at some point in their lifetime, although estimates such as these vary widely across studies [10]. Studies have also documented overlapping symptoms in the 2 conditions, including attention difficulties, cognitive rigidity, behavioral challenges, social communication impairments, and heightened sensory sensitivity [10,12-14]. Despite these advances, the relationship between the 2 conditions remains a lively topic of research interest.

### Public Attention to ADHD and Autism

The neurodiversity movement emerged from autistic communities in the 1990s and has gained substantial momentum over the past decade, largely through online autistic self-advocacy and protest [15-17]. In this paper, “autistic communities” refers specifically to groups led by and primarily composed of autistic individuals. In contrast, “autism communities” include a broader network, encompassing nonautistic parents of autistic children, professionals, and other support stakeholders. The concept of neurodiversity posits conditions such as autism and ADHD as natural variations in human cognitive functioning rather than deficits that need to be fixed [18,19]. The amplification of autistic voices and perspectives, alongside the traction of the neurodiversity concept and movement, has led to emerging debates that have had increasing influence on academic, clinical, and public understandings of autism, ADHD, and related neurodevelopmental conditions [17]. However, these debates have been largely adult-focused (“autistic discourse”), which has led to some tension with the advocacy perspectives of parents of autistic children (“autism discourse”) [17]. Regardless, its influence on public understanding and awareness has contributed to increased recognition of neurodevelopmental conditions in adulthood, which has been attributed to the growing demand for diagnostic assessments among adult populations [20].

Neurodiversity advocacy has drawn public attention to the frequent co-occurrence of ADHD and autism, while emphasizing the importance of lived experience over clinical definitions. Social media has enabled neurodivergent communities to develop frameworks and concepts for understanding these conditions [21]. For example, neurodivergent communities have proposed features of ADHD and autism that extend beyond formal diagnostic criteria, such as sensory sensitivity and rejection sensitivity dysphoria—the latter describing intense emotional responses to perceived criticism and rejection, though not currently recognized in diagnostic manuals [22]. Additionally, the term “AuDHD” was coined to describe the distinct experiences and challenges of individuals with both conditions [22]. While research is beginning to examine experiences and perspectives of co-occurring ADHD and autism and how they may differ from clinical models [22,23], the impact of this increased attention on public perceptions remains understudied. While ADHD and autism remain conceptually distinct in official diagnostic systems such as *DSM-5*, heightened public attention may be emphasizing their perceived similarities and blurring their boundaries. While this trend may help individuals to identify their experiences as evidence of autism or ADHD, thereby reducing underdiagnosis, it could also foster overdiagnosis in cases where 1 condition occurs in the absence of the other.

### Related Work

The rise of social media has provided opportunities for researchers to study mental illness–related discourse. Machine learning and natural language processing techniques enable the extraction of language features for predicting or detecting mental health conditions [24]. A frequently used technique is topic modeling, an unsupervised learning method that identifies patterns in broader ideas or sentiments across unlabeled social media posts. For instance, researchers have applied topic modeling to understand experiences associated with depression [25], eating disorders [26], and schizophrenia [27]. Guntuku et al [28] analyzed X (previously known as Twitter) posts from users who self-reported ADHD diagnoses and identified recurring themes such as difficulties with focus and self-regulation, emotional dysregulation, self-criticism, substance use, and exhaustion. Although most research in this area has examined discourse about mental health conditions at a single point in time, several studies have explored diachronic changes in discourse. Studies based on large historical text corpora have shown that mental health–related concepts such as “anxiety” and “depression” [29], as well as broader terms such as “mental illness” and “mental health” [30], have undergone significant semantic shifts over time. For example, Vylomova and Haslam [31] found that the concept of “trauma” broadened its meaning in academic psychology texts from the 1970s to the 2010s. While semantic shifts are often rapid in dynamic environments such as social media and online communities [32], examining changes of mental health-related concepts in these contexts remains underexplored.

Most research using social media data has focused on individual mental health conditions rather than exploring relationships between them, and the few that have done so have taken a cross-sectional approach. For instance, Park et al [33] applied textual cluster analysis to Reddit subreddits focused on anxiety, depression, and posttraumatic stress disorder (PTSD), finding that the anxiety and PTSD subreddits were more similar to each other than to the depression subreddit. Kalantari et al [34] examined neurodiversity discourse on Reddit by comparing the ADHD subreddit and the autism subreddit between 2018-2020 using a topic modeling approach. To assess overlapping topics, they calculated document vector similarity across all pairs of topics between the 2 subreddits, finding that common topics included diagnosis, treatment (eg, medication), school, work, and social interactions. No studies to date have examined temporal changes in the relationships between mental health and neurodivergent conditions. Understanding how relationships among conditions have changed could shed light on shifting perceptions of how mental health conditions overlap and interact, in ways that may not align with traditional clinical diagnostic categories, helping to explain the growing trend of individuals self-diagnosing and identifying with multiple diagnostic labels [35].

### This Study

This study aims to characterize and understand shifts in the relationship between ADHD and autism during a period of increasing public attention to these conditions. There has been growing engagement with mental health–related discussions on social media, particularly in recent years [36], and platforms such as Reddit provide a valuable data source for exploring shifts in public perceptions. Using posts from the largest ADHD and autism subreddits, r/ADHD and r/autism, spanning 2012 to 2022, we address three research questions: (1) How have the frequency of combined mentions of the two conditions and overlapping users of the two subreddits changed over time? (2) How has the semantic similarity between discourse on ADHD and autism changed over time? (3) How have the topics discussed in r/ADHD and r/autism shifted over time? For the first 2 questions, we predicted that the discourse on ADHD and autism would increasingly converge in terms of comentions, user overlap, and semantic similarity, whereas the third question was exploratory. Our methodology provides a framework that not only identifies trends but also explores their underlying drivers. By applying multiple natural language processing techniques to data drawn from the period 2012 to 2022, we provide a basis for future studies aiming to monitor and interpret shifts in how the public conceptualizes relationships among mental health conditions.

## Methods

### Dataset

Due to its large volume of user-generated content, Reddit is a valuable platform for studying online mental health and neurodivergence-related discourse. Reddit is a forum-style social media platform where online communities, known as “subreddits,” are organized around specific topics or interests. Each subreddit is dedicated to discussions on its subject, with subreddit names prefixed by “r/” (eg, r/funny, r/worldnews, and r/gaming). Within each subreddit, users can submit posts to initiate discussions, while comments are responses to these posts or other comments, enabling conversation and interaction.

Demographically, Reddit users in the United States tend to skew male, younger (18–29 years old), with higher income levels and education (typically college-educated. Most reside in urban or suburban areas and exhibit a slight left-leaning political orientation. Racially, Reddit users are more likely to identify as Asian, followed by Hispanic and White, with Black users being the smallest demographic group [37].

**Figure 1:**
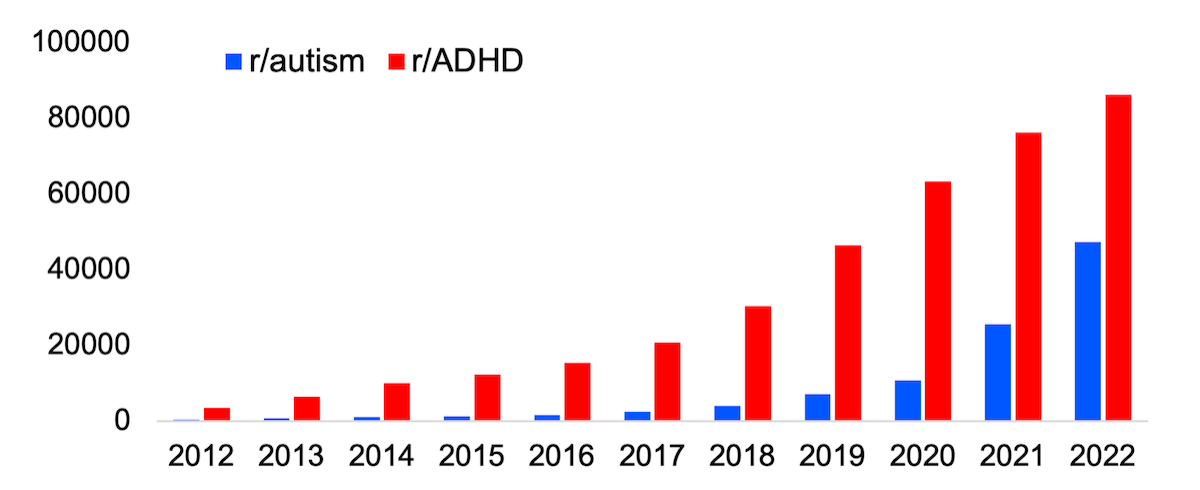
Total number of posts per year in r/autism and r/ADHD from 2012 to 2022. ADHD: attention-deficit/hyperactivity disorder.

Our dataset was incrementally collected from Pushshift.io until the beginning of 2023. Therefore, the dataset ranged between the years of 2012 to 2022. The portion of the dataset that we focused on included posts from r/autism and r/ADHD. These 2 were selected for their size and longevity as subreddits dedicated to discussions of ADHD and autism, in contrast to more specific subreddits such as r/adhdwomen or r/AutisticAdults. We included a total of 372,355 posts from r/ADHD and 106,560 posts from r/autism. The post counts per year for each subreddit are shown in Figure 1.

### Ethical Considerations

This study was approved by the University of Melbourne Human Research Ethics Committee (2024-30416-58642-3). The research involved analysis of publicly available social media data and was conducted following institutional guidelines and ethical standards for studies involving human participants. A waiver of informed consent was granted due to the nature of the data. Analyses were conducted on aggregated data only, and no personally identifiable information or quotations are reported in this paper. No compensation was provided to individuals, as the research did not involve the recruitment or participation of identifiable persons.

### Data Analysis

To assess the rate of comentions, we analyzed the frequency distribution of ADHD mentions in r/autism and autism mentions in r/ADHD across time. Though our focus is on ADHD and autism mentions, we also examined comentions of other common comorbid conditions—obsessive-compulsive disorder (OCD), PTSD, bipolar disorder—to determine the extent to which trends were specific to our focal conditions. To illustrate general trends, we only used the most commonly recognized terms for each of these conditions and did not include other terms used to refer to them. The relative frequency for each condition was calculated by dividing the number of posts mentioning the condition by the total number of posts in that subreddit for each year. We also assessed changes in user overlap between the 2 subreddits to capture shifts in cross-subreddit discussions. In this analysis, relative frequency was calculated by dividing the number of unique users who posted in both subreddits by the total number of unique users for each year.

To evaluate the semantic similarity between ADHD and autism discourses, as well as their relationship to the other common comorbid conditions over time, a Word2Vec model [38] was used to generate word embeddings, which are dense vector representations of words in a continuous vector space. Following the approach of Hamilton et al [39] for analyzing semantic change, we trained separate Word2Vec models for each subreddit, using a minimum word count of 20, a context window of 10, and 200-dimensional embeddings. These models were created for each year from 2018 to 2022, as there were insufficient posts to yield reliable similarity estimates before 2018, particularly in r/autism.

To enable meaningful temporal comparisons, we aligned the models across years using the orthogonal Procrustes method, which maintains consistent axes and preserves the approximate directions and magnitudes of word embeddings across vector space [38]. Each yearly model was aligned to the 2022 model (the most recent year in our dataset) to track changes in the semantic relationship between “ADHD” and “autism” over time. We then calculated cosine similarity—the cosine of the angle between word vectors—as a measure of semantic relatedness between these terms in each year.

A topic modeling approach was used to examine themes in ADHD and autism discourse and how they changed over time. The dataset was divided into 2 periods: 2012-2019 and 2020-2022. We elected to compare topics between 2 distinct multiyear periods, rather than dividing the dataset by year, as a simple 2-way comparison was likely to yield more interpretable results. We used BERTopic [40], a topic-modeling algorithm that has demonstrated strength in handling complex, context-rich social media data [41]. BERTopic leverages pretrained BERT models, specifically Sentence BERT, to encode each post into high-dimensional contextual embeddings. Then we apply dimensionality reduction to the embeddings using the uniform manifold approximation and projection (UMAP) algorithm [42]. Following this, we use the hierarchical density-based spatial clustering of applications with noise (HDBSCAN) algorithm [43] to identify distinct clusters within this transformed space, without having to predefine the number of clusters. The model then assigns each document to a single topic by calculating the cosine similarity between the document’s embedding and the centroid of each topic cluster. After extracting the topics, the most relevant terms and representative posts (ie, posts closest to the topic’s centroid) for each topic were analyzed and manually assigned an appropriate label.

## Results

**Figure 2:**
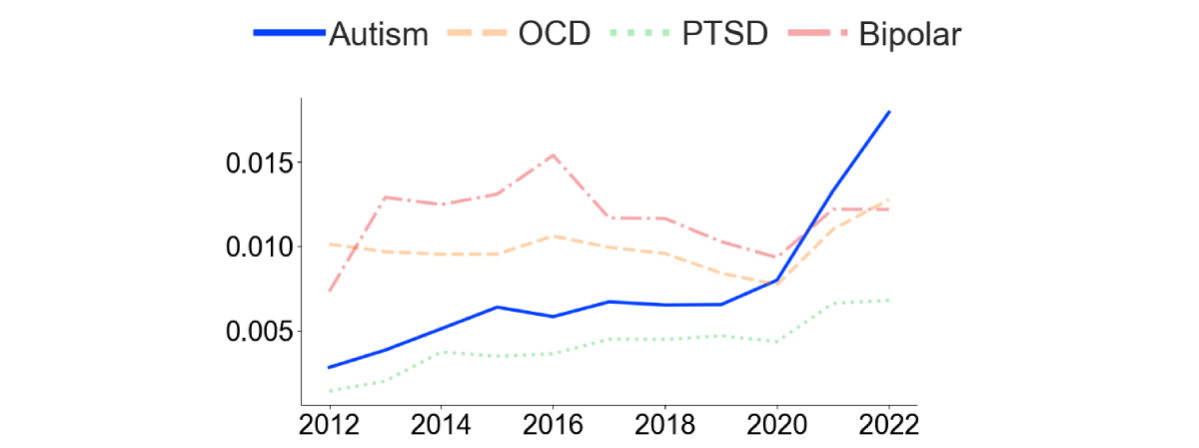
Relative frequency of posts mentioning autism, OCD, PTSD, and bipolar from 2012 to 2022 in r/ADHD. Relative frequency was calculated by dividing the frequency per year by the total number of posts per year. ADHD: attention-deficit/hyperactivity disorder; OCD: obsessive-compulsive disorder; PTSD: posttraumatic stress disorder.

**Figure 3:**
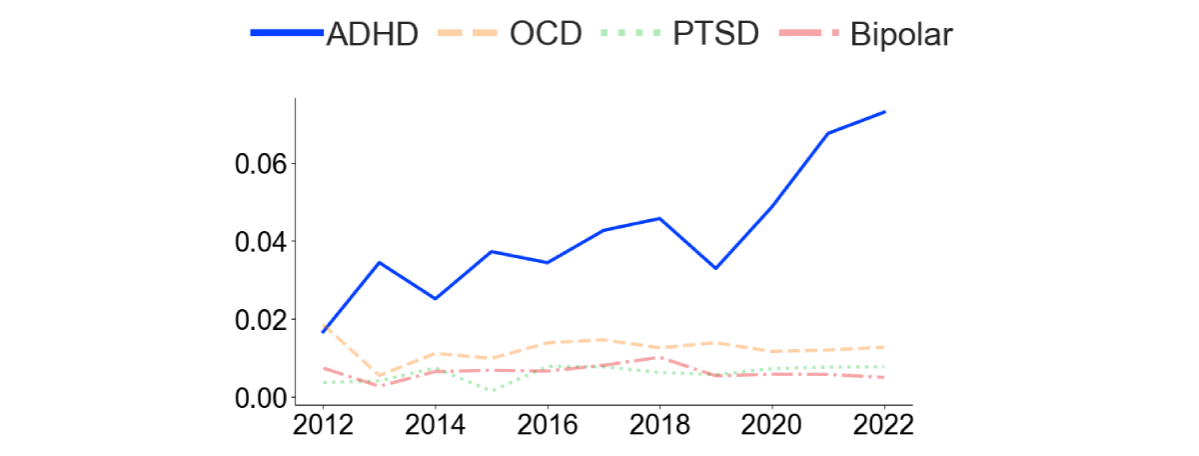
Relative frequency of posts mentioning ADHD, OCD, PTSD, and bipolar from 2012 to 2022 in r/autism. Relative frequency was calculated by dividing the frequency per year by the total number of posts per year. ADHD: attention-deficit/hyperactivity disorder; OCD: obsessive-compulsive disorder; PTSD: posttraumatic stress disorder.

**Figure 4:**
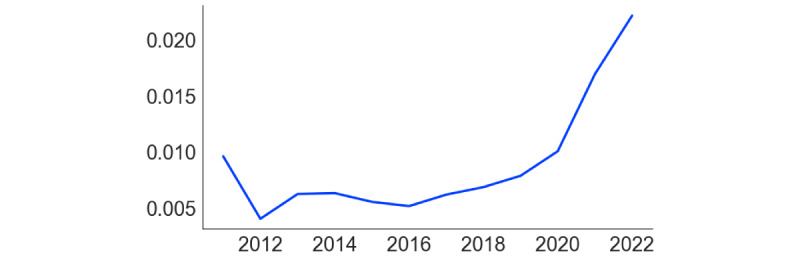
Relative frequency of user overlap in r/autism and r/ADHD from 2012 to 2022. Relative frequency was calculated by dividing the frequency of unique overlap of users per year by the total unique users from that year.

Figures 2 and 3 present the analysis of comentions of autism and ADHD in the 2 subreddits. In r/ADHD (Figure 2), the proportion of posts mentioning “autism” increased steadily over time, with a steep rise starting in 2021. Before 2021, “bipolar” and “OCD” were the most frequently mentioned additional conditions in the subreddit, but in that year, “autism” took the lead. In r/autism (Figure 3), the proportion of posts mentioning “ADHD” also increased over time. In contrast, the frequency of posts mentioning OCD, PTSD, and bipolar remained stable and low. Figure 4 demonstrates that the rate of overlap of users between r/autism and r/ADHD grew over time, with a steep increase beginning in 2020. Thus, consistent with our prediction regarding our first research question, each condition increasingly featured in the other’s subreddit, and a growing proportion of users contributed to both.

**Figure 5:**
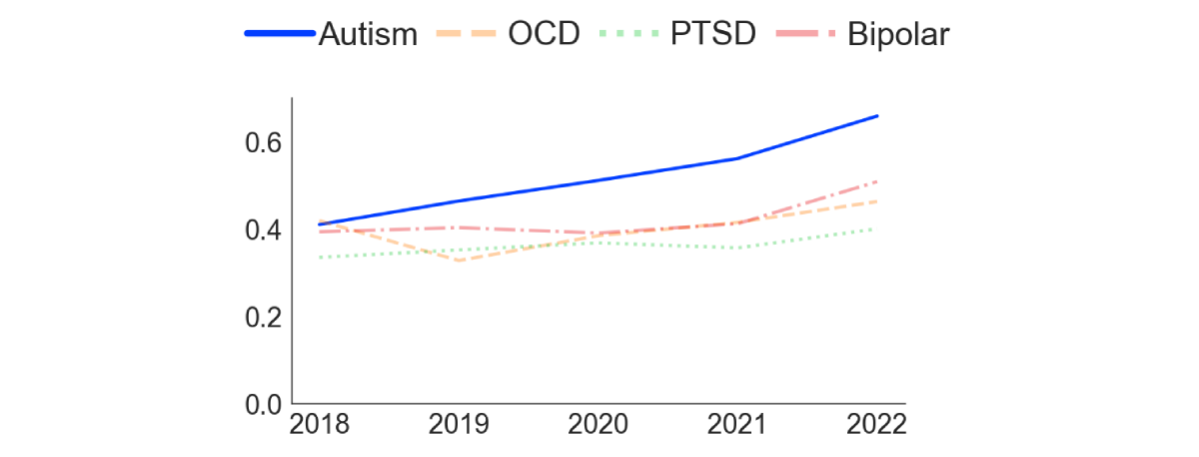
Semantic similarity between autism, OCD, PTSD, and bipolar disorder with ADHD in r/ADHD from 2018 to 2022. The changes in semantic similarity were calculated by measuring cosine similarity between Word2Vec embeddings of ADHD and the other conditions. Higher values indicate greater contextual association in subreddit discussions. ADHD: attention-deficit/hyperactivity disorder; OCD: obsessive-compulsive disorder; PTSD: posttraumatic stress disorder.

**Figure 6:**
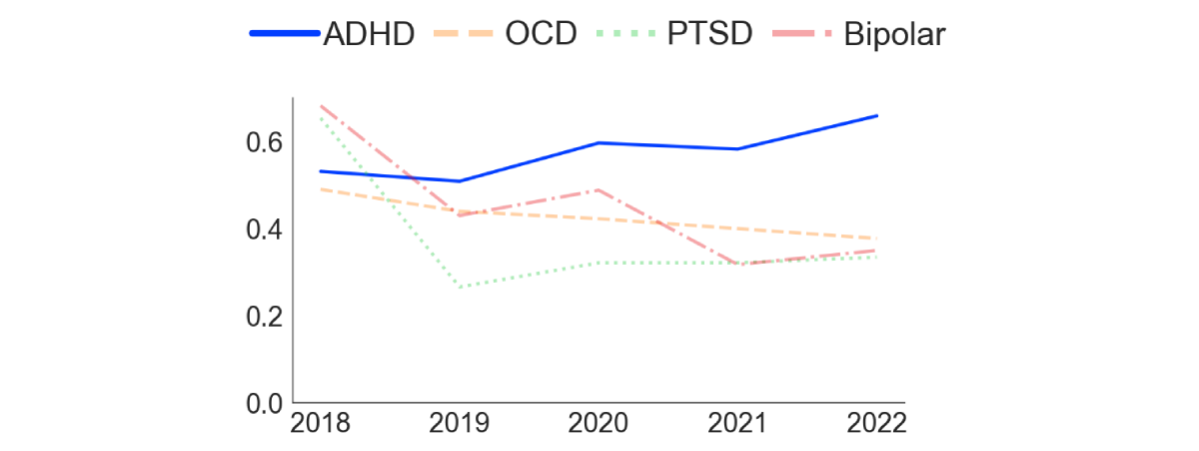
Semantic similarity between ADHD, OCD, PTSD, and bipolar with autism in r/autism from 2018 to 2022. The changes in semantic similarity were calculated by measuring cosine similarity between Word2Vec embeddings of autism and the other conditions. Higher values indicate greater contextual association in subreddit discussions. ADHD: attention-deficit/hyperactivity disorder; OCD: obsessive-compulsive disorder; PTSD: posttraumatic stress disorder.

Figures 5 and 6 indicate that the semantic similarity of “ADHD” and “autism,” assessed by the cosine similarity of their semantic vectors, has increased over time in both subreddits. The increase is steeper in r/ADHD (Figure 5) than in r/autism (Figure 6), implying that Reddit discourse about ADHD has aligned with autism to a greater degree than the reverse. From 2019 onward, the 2 conditions were semantically closer to one another than any of the other commonly mentioned conditions, most of which became less semantically related or held steady over the 2018-2022 study period. Consistent with our prediction regarding the second research question, these findings indicate a growing convergence in the contextual use of “ADHD” and “autism” within the 2 subreddits over time.

**Figure 7:**
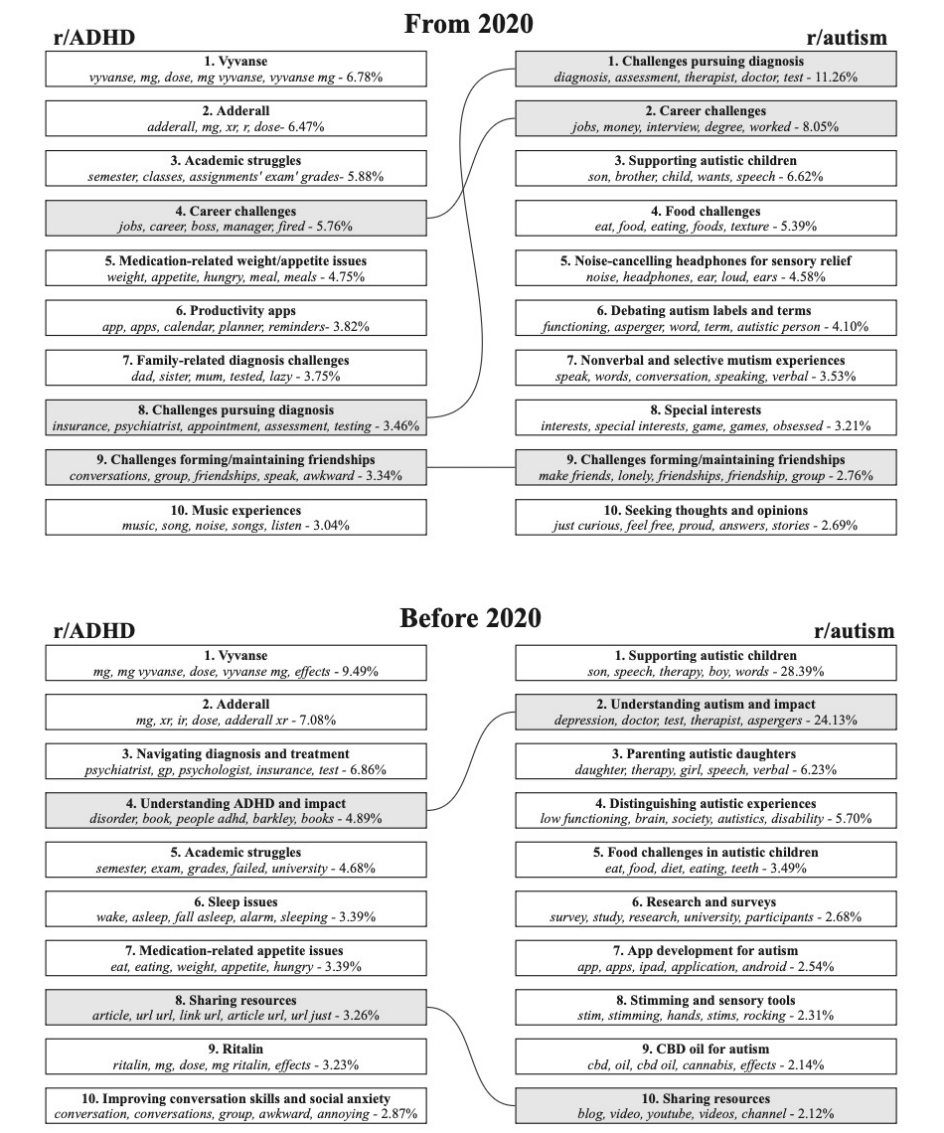
Topic modeling results showing topic names, most relevant topic words, and post percentages for pre-2020 (top) and 2020-onward (bottom) posts. The results are split for r/ADHD (left) and r/autism (right), with shared topics highlighted for comparison. ADHD: attention-deficit/hyperactivity disorder; CBD: cannabidiol.

Figure 7 summarizes the topic modeling analyses from 2020-2022 and 2012-2019, respectively. The figure reveals a considerable change in the topics discussed in the 2 forums before and after 2020, likely due to growing membership, emerging research, and broader social trends. Nevertheless, there are some temporally consistent topics. In r/ADHD, consistent topics across both periods related to: medication-related issues (topics 1, 2, 7, and 9 before 2020 and topics 1, 2, and 5 from 2020), diagnosis-related challenges (topic 3 before 2020 and topics 8 and 7 from 2020), interpersonal challenges (topic 10 before 2020 and topic 9 after 2020), and school and college struggles (topic 5 before 2020 to topic 3 from 2020).

Topics discussed in r/autism were less consistent across the 2 periods (Figure 7). Discussions related to supporting autistic children were consistent across both periods and represented 38% (4179/10,967) of posts included in the topic modelling analysis before 2020 (topics 1, 3, and 5), although this reduced to 7% (2896/43,770) of posts from 2020 (topic 3). Discussions related to food challenges were also consistent across both periods (topic 5 before 2020 and topic 4 from 2020). However, before 2020, these discussions primarily focused on the experiences of autistic children, whereas from 2020, these shifted to focus on users’ personal experiences. These trends suggest that r/autism has become more focused on adult experiences over time.

In r/ADHD (Figure 7), notable shifts in discussion topics were observed between the 2 periods. Three topics were unique to the pre-2020 period: understanding ADHD (topic 4), resource sharing (topic 8), and sleep issues (topic 6). From 2020 onward, 3 new topics emerged: career challenges (topic 4), productivity apps (topic 6), and music experiences (topic 10). The emergence of career challenges (topic 4) and productivity apps (topic 6) indicates a growing emphasis on adult professional life within the r/ADHD subreddit.

There were more changes in topics over time in r/autism compared to r/ADHD (Figure 7). Before 2020, topics relating to understanding autism (topic 2), distinguishing autistic experiences (topic 4), research and surveys (topic 6), app development for autism (topic 7), stimming and sensory tools (topic 8), cannabidiol oil for autism (topic 9), and resource sharing (topic 10) were observed. From 2020 onward, new topics emerged, including challenges pursuing diagnosis (topic 1), employment challenges (topic 2), noise-canceling headphones for sensory relief (topic 5), debating autism labels and terms (topic 8), challenges forming and maintaining friendships (topic 9), and seeking thoughts and opinions (topic 10).

Over time, the shared topics between r/ADHD and r/autism changed in nature and increased in number (Figure 7). Before 2020, both subreddits shared a topic on understanding their respective conditions (topic 4 in r/ADHD and topic 2 in r/autism) and a topic focused on sharing resources (topic 8 in r/ADHD and topic 10 in r/autism). From 2020, a total of 3 different shared topics emerge: challenges maintaining meaningful relationships (topic 9 in r/ADHD and topic 9 in r/autism), challenges in professional life (topic 4 in r/ADHD and topic 2 in r/autism), and diagnosis challenges (topic 8 in r/ADHD and topic 1 in r/autism). This pattern suggests an increasing convergence in the topics discussed between the 2 subreddits over time, consistent with the increased convergence observed in comentions, users, and contextual meaning.

## Discussion

### Principal Findings

This study is the first to systematically examine changes in public discourse surrounding ADHD and autism. Previous research has predominantly focused on the clinical or epidemiological dimensions of the relationship between ADHD and autism [2] and has largely overlooked the insights that can be drawn from naturalistic social media data. Using our novel methodology, we captured multiple dimensions of the relationship between ADHD and autism, providing insights into both the changes themselves and their potential drivers. By analyzing temporal patterns in r/ADHD and r/autism, we found that these conditions have become progressively more associated based on frequency of comentions, semantic similarity, user overlap, and discussion themes. Taken together, our findings clarify how discourse around these conditions has converged during a period when they have attracted rising public attention [22,44].

Findings relevant to our first research question revealed that over the past decade, ADHD has become increasingly prominent in autism-related discourse, while autism has similarly gained prominence in ADHD-related discussions. The overlap in users of the 2 subreddits has also grown substantially. Findings relevant to our second research question indicated that “ADHD” and “autism” have become increasingly used in similar semantic contexts. That is, ADHD and autism have not only become more associated through their rising salience within each other’s discourse but have also grown increasingly related in meaning. Together, these trends may reflect a broader cultural shift toward understanding ADHD and autism as interconnected rather than distinct conditions. This shift could be attributed to the growing influence of neurodiversity advocacy, which has played a large role in emphasizing the affinities between these conditions [22].

Findings addressing our third research question revealed that discussions within both subreddits have changed over time and become increasingly focused on the experiences of adults. This is evident in r/ADHD, for instance, through the emergence of topics related to career challenges and the use of productivity tools. Additionally, in r/autism, there was a noticeable decline in discussions related to support for autistic children, and an increased focus on adult autistic experiences (such as food-related and professional life challenges). These shifts point to a growing view that the 2 conditions pose lifelong challenges rather than being restricted to childhood. Additionally, it demonstrates the influence of neurodiversity discourse, which tends to center on adult experiences [16,17].

The topic modeling analysis suggests that the growing convergence of the 2 subreddits was not only due to their emerging shared focus on themes relevant to adults but also in new shared foci on seeking formal diagnoses and on managing interpersonal relationships. The former theme may reflect the rising demand for ADHD and autism assessments among adults [45]. The latter suggests that relational challenges are being increasingly associated with both conditions. As the emerging interest in rejection sensitivity dysphoria within the neurodivergent community shows, conditions that were once conceptualized as primarily cognitive in nature have acquired more social and emotional conceptualizations in recent years. This finding contributes to the emerging debate on how lived experiences of co-occurring ADHD and autism diverge from traditional clinical frameworks, particularly for historically overlooked groups such as women [22,23].

### Limitations

To our knowledge, this study is the first to investigate shifts in discursive relationships between mental health conditions using social media data. Future research should extend this approach to examine changes in the relationships between other conditions, such as anxiety and depression, or PTSD and eating disorders. These investigations should focus on conditions that have garnered growing public attention, given the increasing interest in understanding the risks and benefits associated with the rising prominence of mental health information on social media [35].

Despite its novel focus, this study has several limitations. One is its reliance on data from only 2 subreddits, albeit large and prominent ones, which may not fully capture the diversity of experiences and perspectives related to ADHD and autism. Future research could incorporate a broader range of subreddits, such as r/AutisticAdults or r/adhdwomen, to ensure greater representation of varying groups and experiences.

Second, while social media data provides access to large-scale, naturalistic public discourse, a small subset of users generates most content, while most users do not actively contribute [46], thus limiting the generalizability of our findings. This limitation is further compounded by the demographic skew of Reddit data [37]. To address these issues, future studies could adopt traditional methods such as surveys with more representative samples or extend similar methodologies to other platforms such as X, Facebook, or TikTok to determine whether the observed trends hold across platforms and in offline contexts.

Finally, the dataset used in this study extends only until 2022, potentially missing recent developments in public discourse. The decade-long timespan covered by the dataset nevertheless allows for meaningful insights into broad trends over an extended period in which public understandings of mental health and neurodiversity underwent significant change. To enhance the relevance of findings, future research should incorporate datasets with more recent data. This is particularly important given the fast pace of semantic change that occurs on social media and online communities [32]. Nevertheless, our findings demonstrate a promising approach to mapping and understanding these important developments, offering novel insights into the evolving representations of ADHD and autism in the wider culture.

### Conclusions

Our research demonstrates that ADHD and autism have increasingly converged in public discourse during a period of rising public attention. This convergence appears to reflect a growing emphasis on assessment challenges, the lifelong nature of these conditions, and their social-emotional dimensions rather than cognitive presentations. These findings contribute to wider discussions about the impacts of rising public interest in mental health concepts. They imply that public understandings of relationships between conditions are dynamic and changing in ways that diverge from diagnostic frameworks. Future research should continue investigating changing mental health conceptualizations on social media, as these dynamics are becoming increasingly important for the future of psychiatric practice.

## Abbreviations

ADHD: attention-deficit/hyperactivity disorder
*DSM*: *Diagnostic and Statistical Manual of Mental Disorders*
*DSM-III*: *Diagnostic and Statistical Manual of Mental Disorders*, Third Edition
*DSM-IV*: *Diagnostic and Statistical Manual of Mental Disorders*, Fourth Edition
*DSM-5*: *Diagnostic and Statistical Manual of Mental Disorders*, Fifth Edition
HDBSCAN: hierarchical density-based spatial clustering of applications with noise
OCD: obsessive-compulsive disorder
PTSD: posttraumatic stress disorder
UMAP: uniform manifold approximation and projection

## Data Availability

The datasets analyzed during this study are not publicly available due to Reddit’s terms of use.
